# Characterization of the Oral Bacteria in Patients With Neuroendocrine Tumors of the Pancreas

**DOI:** 10.1002/cam4.71840

**Published:** 2026-04-19

**Authors:** Jiayue Yao, Chunming Fei, Han Wu, Zhenyu Zhang, Zongdan Jiang

**Affiliations:** ^1^ Department of Gastroenterology Nanjing First Hospital, Nanjing Medical University Nanjing Jiangsu China; ^2^ Department of Gastroenterology Nanjing Luhe People's Hospital, Yangzhou University Nanjing Jiangsu China; ^3^ Department of Gastroenterology Nanjing Jiangbei Hospital Nanjing Jiangsu China

**Keywords:** 16S rRNA gene sequencing, microbiota, oral bacteria, pNETs

## Abstract

The primary objective of this study was to investigate dysbiosis in the oral microbiota of patients with pancreatic neuroendocrine tumors (pNETs) and to identify potential biomarkers for clinical diagnosis and prognostic evaluation of pNETs. Healthy controls and pNETs patients were recruited from our hospital. Salivary flora were profiled in healthy subjects (HS group) and pNETs patients (PS group) using 16S rRNA gene sequencing. Microbial diversity was assessed by α‐diversity (Tukey test) and β‐diversity (Partial Least Squares Discriminant Analysis, PLS‐DA). Taxonomic differences between groups were evaluated using linear discriminant analysis effect size (LEfSe). The salivary microbiota of pNETs patients showed higher abundance and diversity compared to healthy controls. Dominant bacterial phyla in both groups include *Proteobacteria*, *Firmicutes*, *Bacteroidota*, *Actinobacteriota*, *Fusobacteriota*, *Cyanobacteria*, and *Campilobacterota*. At the genus level, *Leptotrichia*, *Actinobacillus*, and *Granulicatella* were more abundant in the PS group. LEfSe analysis further indicated a greater abundance of *Rothia*, *Chloroplast*, *Leptotrichia*, *Actinomyces*, and *Granulicatella* in the PS group. Our findings offer initial evidence suggesting a potential link between oral microbiome dysbiosis and pNETs, and identify microbial features that could be evaluated in future studies as potential biomarkers for clinical diagnosis and prognosis.

**Abbreviations**
GEP‐NETs: gastroenteropancreatic NETs
KEGG: Kyoto Encyclopedia of Genes and Genomes
LDA: linear discriminant analysis
LEfSe: Linear discriminant analysis effect size
NETs: Neuroendocrine neoplasms
OTUs: operational taxonomic units
PCoA: principal coordinates analysis
PICRUSt: Phylogenetic Investigation of Communities by Reconstruction of Unobserved States
PICRUSt: Phylogenetic Investigation of Communities by Reconstruction of Unobserved States
PLS‐DA: Partial Least Squares Discriminant Analysis
pNETs: pancreatic neuroendocrine tumors
SRA: Sequence Read Archive
WHO: World Health Organization

## 1 Introduction

Neuroendocrine neoplasms (NETs) comprise a heterogeneous group of tumors originating from secretory cells of the diffuse neuroendocrine system. These tumors typically display a relatively indolent growth pattern and can secrete various peptide hormones and biogenic amines. Although NETs may occur at any age, their incidence is higher in individuals aged 50 years and above. Among gastroenteropancreatic NETs (GEP‐NETs), two main subtypes are recognized: Carcinoid tumors of the gastrointestinal tract and pancreatic neuroendocrine tumors (pNETs) [1].

Early detection and diagnosis of NETs primarily depend on endoscopy, imaging techniques, and pathological examination. Historically, the diagnosis rate of GEP‐NETs has been low, likely due to their widespread distribution, nonspecific clinical symptoms, and limited clinical awareness. However, with advances in diagnostic methods, widespread use of endoscopy, and continuous improvements in imaging technology, the global incidence of NETs has risen notably in recent years [2]. The overall 5‐year survival rate for pNETs is approximately 80% across all stages, ranging from 60%–100% for localized disease, 40% for regional disease, and 29% for distant metastasis [3, 4]. Moreover, long‐term disease‐specific survival exceeds 50% even 20 years after surgical resection of pNETs [5]. Prognostic factors such as tumor size, grade, metastatic progression, and lymph node involvement significantly contribute to the poor outcomes associated with pNETs. Therefore, early detection is crucial for improving survival and prognosis in pNET patients.

As the entrance to the human digestive tract, the oral cavity hosts a diverse microbial community comprising over 700 bacterial species [6]. Changes in the composition of this microbiota can disrupt its functional balance and substantially impact overall health [7]. Oral microorganisms are not only implicated in periodontal diseases but are also increasingly recognized as potential contributors to systemic conditions.

Previous research has suggested a potential involvement of the oral microbiota in the development of pancreatic cancer. Biologically, oral bacteria may reach the pancreas through hematogenous spread or via an oral‐gut‐pancreas axis, while certain pathogens may directly influence disease pathogenesis by invading host cells, disrupting cytokine activity, and interfering with cellular signaling pathways [8, 9, 10].

Although pNETs and pancreatic cancer differ in origin and behavior, they share anatomical location and aspects of the tumor microenvironment. Despite this overlap, the relationship between the oral microbiota and pNETs remains largely unexplored.

Therefore, we hypothesized that pNETs patients harbor a distinct oral microbial community, the compositional features of which may be linked to disease presence and progression. To test this, we conducted 16S rRNA gene sequencing, systematically comparing the salivary microbiome of pNETs patients and healthy controls. Our findings may provide new insights into the potential role of the oral microbiota in pNETs pathogenesis.

## 2 Materials and Methods

### 2.1 Study Participants

Between November 2019 and July 2020, a total of fifteen patients with pancreatic neuroendocrine tumors (pNETs), constituting the PS group, and fifteen healthy control volunteers (HS group) were prospectively recruited from our hospital. The diagnosis of pNETs was pathologically confirmed for all patients in the PS group. Eligibility for the HS group required no history of organic, systemic, or oral diseases and a negative family history of tumors.

The diagnosis of pancreatic neuroendocrine tumors in patients was confirmed through pathological examination. The specific criteria for inclusion are as follows: (1) Pathological diagnosis of pancreatic Neuroendocrine tumor; (2) Once received intervention measures, but didn't receive any treatment in the last month, including surgery, chemotherapy, biological agents or targeted therapy; while the healthy control individuals were selected based on their absence of organic diseases, systemic diseases, oral diseases, and family history of tumors. The specific criteria for exclusion are as follows: (1) Have a history of digestive system diseases such as peptic ulcer, irritable bowel syndrome, inflammatory bowel disease, and malignant tumor of digestive tract; (2) Have a history of metabolic diseases such as diabetes and fatty liver; (3) Have a history of upper respiratory tract infection, intestinal infection in the past week; (4) Have a history of oral infection in the past month, such as periodontitis; (5) Patients with neuropsychiatric disorders cannot cooperate with this experiment; (6) Take PPI, gastrointestinal motility drugs, anticoagulants, antibiotics and other drugs in recent 2 weeks; (7) Pregnant and lactating women and patients with severe cardiopulmonary dysfunction; (8) Smoking.

The study protocol was approved by the institutional review board of Nanjing First Hospital, and all experimental procedures were conducted in strict adherence to the approved guidelines and regulations.

### 2.2 Sample Collection

Several measures were taken to minimize potential interference from food residues during saliva collection. Participants were instructed to fast for at least 4 h and refrain from smoking and alcohol consumption prior to the procedure. In accordance with a standardized protocol, each participant rinsed their mouth with water for about 10 s, repeating the process 2–3 times. Approximately 3–4 mL of saliva was collected from each individual using pre‐labeled collection cups. The samples were immediately placed on ice, transported to the laboratory, and stored at −80°C for subsequent DNA extraction.

To minimize technical batch effects, all saliva samples were stored at −80°C immediately after collection until the entire sample set was completed. Genomic DNA was then extracted from all samples in a single, consolidated batch. The resulting DNA libraries were prepared simultaneously using the same lot of reagents in one session and were subsequently pooled and sequenced in a single run. This approach ensured that any procedural variability was applied uniformly across all samples, thereby reducing technical confounders.

### 2.3 DNA Extraction

The extraction of total genomic DNA from the samples was carried out using the QIAamp DNA Mini Kit, employing the bead‐beating method. The DNA concentrations of the extracted samples were standardized to 50 ng/μl to ensure consistency for the subsequent analysis of 16S rDNA genes. The bacterial DNA samples were then stored at −80°C to preserve their integrity for future sequencing studies.

### 2.4 PCR Amplification

The amplification of 16S rDNA genes within the V3‐V4 region was performed using universal primers, specifically 338F (5′‐ACTCCTACGGGAGGCAGCAG‐3′) and 806R (5′‐GGACTACHVGGGTWTCTAAT‐3′). All PCR reactions, encompassing denaturation, annealing, and elongation steps, were conducted using the Phusion High‐Fidelity PCR Master Mix. Following electrophoresis of the PCR products, samples exhibiting a distinct and prominent band between 400 and 450 bp were selected for subsequent mixing and purification, employing the Qiagen Gel Extraction Kit.

### 2.5 Sequencing, Processing, and Analysis

The purified amplicons were pooled in equimolar concentrations and subjected to paired‐end sequencing using an Illumina Novaseq6000 PE250 platform. To ensure data quality, the raw sequences were processed and filtered using QIIME (v1.8.0). Reads that were dereplicated or shorter than 150 bp were removed from further analysis. The remaining filtered reads were then clustered into operational taxonomic units (OTUs) based on a 97% similarity threshold. Taxonomic classification of each OTU was performed by comparing the sequences with the SILVA database (version 138). The raw sequencing data have been deposited in the NCBI Sequence Read Archive (SRA) database with the accession number PRJNA984637.

### 2.6 Statistical Analysis

Statistical analysis was conducted to assess group differences using appropriate methods based on variable characteristics. Continuous variables were analyzed using independent *t*‐tests, White's nonparametric *t*‐tests, or Mann–Whitney U tests. To control the false discovery rate arising from multiple comparisons, the resulting P‐values were adjusted using the Bonferroni correction. Taxa with an adjusted P‐value < 0.05 were considered statistically significant. α diversity was evaluated using the QIIME software, employing the Shannon index and Simpson index. β diversity was assessed through Partial Least Squares Discriminant Analysis (PLS‐DA) to examine diversity differences among groups. Linear discriminant analysis effect size (LEfSe) with an LDA threshold of 3 was employed to identify microbial taxa associated with different groups. Additionally, the Phylogenetic Investigation of Communities by Reconstruction of Unobserved States (PICRUSt) analysis was used to predict Kyoto Encyclopedia of Genes and Genomes (KEGG) biochemical pathways. Statistical analyses were performed using SPSS V19.0 and STAMP V2.1.3. Graphs were generated using GraphPad Prism version 6.0. A significance level of *p* < 0.05 was considered statistically significant.

## 3 Results

### 3.1 Demographic Characteristics of All Individuals

This study comprised a cohort of 15 patients with pancreatic neuroendocrine tumors (pNETs, PS group) and 15 healthy volunteers (HS group). The demographic characteristics of all participants are summarized in Table 1. The two groups were well‐matched, with no significant differences in sex (*p* = 0.57), age (*p* = 0.54), or BMI (*p* = 0.54). In the PS group, most patients had Grade 2 tumors (87%), presented with metastasis (73%), and had undergone either endoscopy or surgery (80%).

**TABLE 1:** The demographic characteristics of all participants.

Characteristics	Healthy volunteers, *n* = 15	pNETs, *n* = 15
Sex (Male/Female)	4/11	5/10
Age	56.07 ± 9.97	53.53 ± 15.63
BMI (kg/m^2^)	19.95 ± 1.92	20.31 ± 1.31
Grade		
G1		1
G2		13
G3		1
Metastasis		11
Treatment		
Endoscopy/surgery		12
Medical treatment		3

### 3.2 Quality Control and Basic Analysis

**FIGURE 1:**
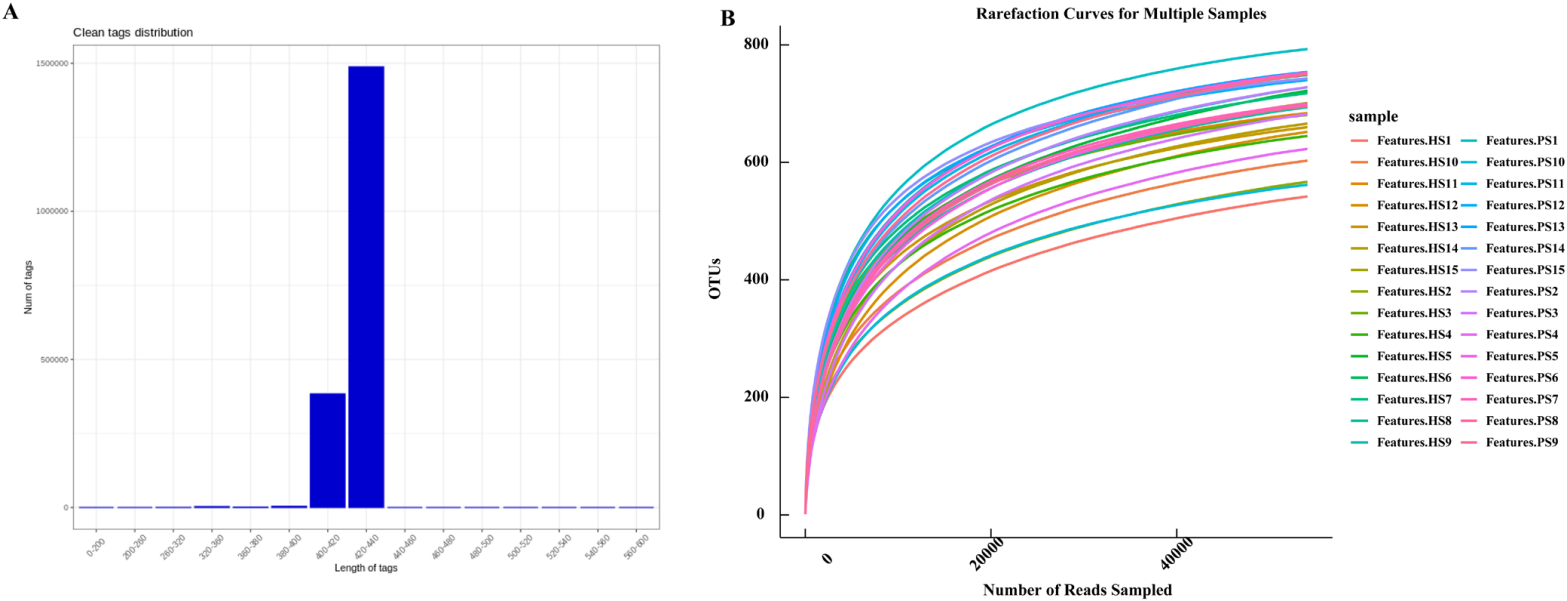
Quality control and basic analysis. (A) The x‐axis represents the sequence length of tags, while the y‐axis represents the number of tags. (B) Rarefaction Curves for Multiple Samples.

After sequencing and implementing quality filtering, our analysis yielded a comprehensive dataset consisting of more than 1.8 million tags, resulting in the identification of a total of 1129 operational taxonomic units (OTUs). It is noteworthy that most tags displayed a prominent length distribution falling within the range of 400–440 bp, as depicted in Figure 1A. The rarefaction curves, as illustrated in Figure 1B, demonstrated a positive correlation between the number of sequences and the number of OTUs, indicating a tendency toward reaching a plateau stage. This finding suggests that the sequencing depth achieved for each sample was sufficient and reliable (Figure 1B).

### 3.3 Microbial Diversity and Richness Between the Two Groups

**FIGURE 2:**
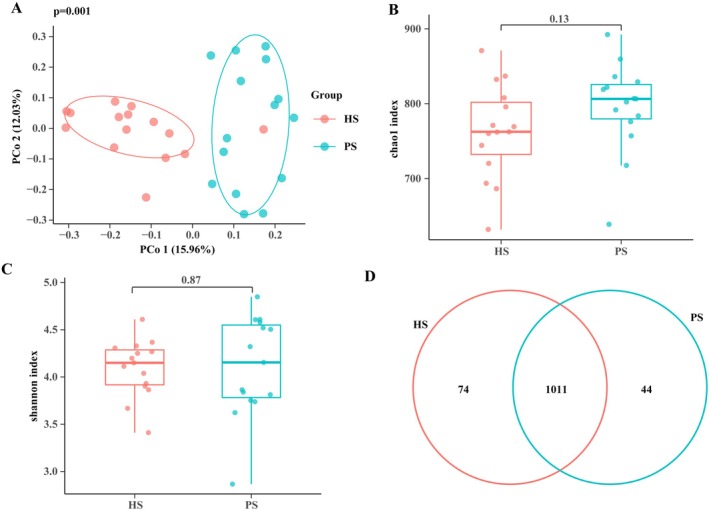
The microbial α diversity and β diversity analysis in two groups. PS group: Pancreatic neuroendocrine tumors; HS group: Healthy volunteers. (A) PLS‐DA revealed different microbial community structures in the two groups. (B) The Chao 1 index was slightly higher in the PS group than in the HS group. (C) The pancreatic neuroendocrine tumors in the PS group had a slightly higher Shannon index in comparison with the HS group. (D) A Venn diagram displayed the overlaps between the two groups.

To systematically investigate the influence of pNETs on the oral microbiome, we first assessed overall structural differences in microbial communities between HS and PS using β‐diversity analysis. Principal coordinates analysis (PCoA) based on Bray–Curtis distances revealed a clear separation between HS and PS samples in the ordination space (Figure 2A), indicating a significant association between pNET status and altered global composition of the salivary microbiota.

Following this confirmation of structural divergence, we further discussed whether such reorganization was driven by changes in species richness and evenness within the communities. However, α‐diversity analysis showed that although Chao1 and Shannon indices were slightly higher in the PS group, the differences did not reach statistical significance (Figure 2B,C).

To more precisely resolve compositional relationships between the two groups, we compared operational taxonomic units (OTUs) using a Venn diagram. The results indicated that the two groups shared a large core set of microbial taxa (1011 OTUs), whereas relatively few OTUs were unique to either group (74 in HS, 44 in PS; Figure 2D). Together with the α‐diversity results, these findings collectively indicate that the shift in salivary microbiota in pNETs patients is not primarily driven by the gain or loss of specific taxa but rather by changes in the relative abundance of commonly present species, ultimately leading to marked reorganization of the overall community structure.

### 3.4 Changes in Oral Microbiota Composition Between the Two Groups

**FIGURE 3:**
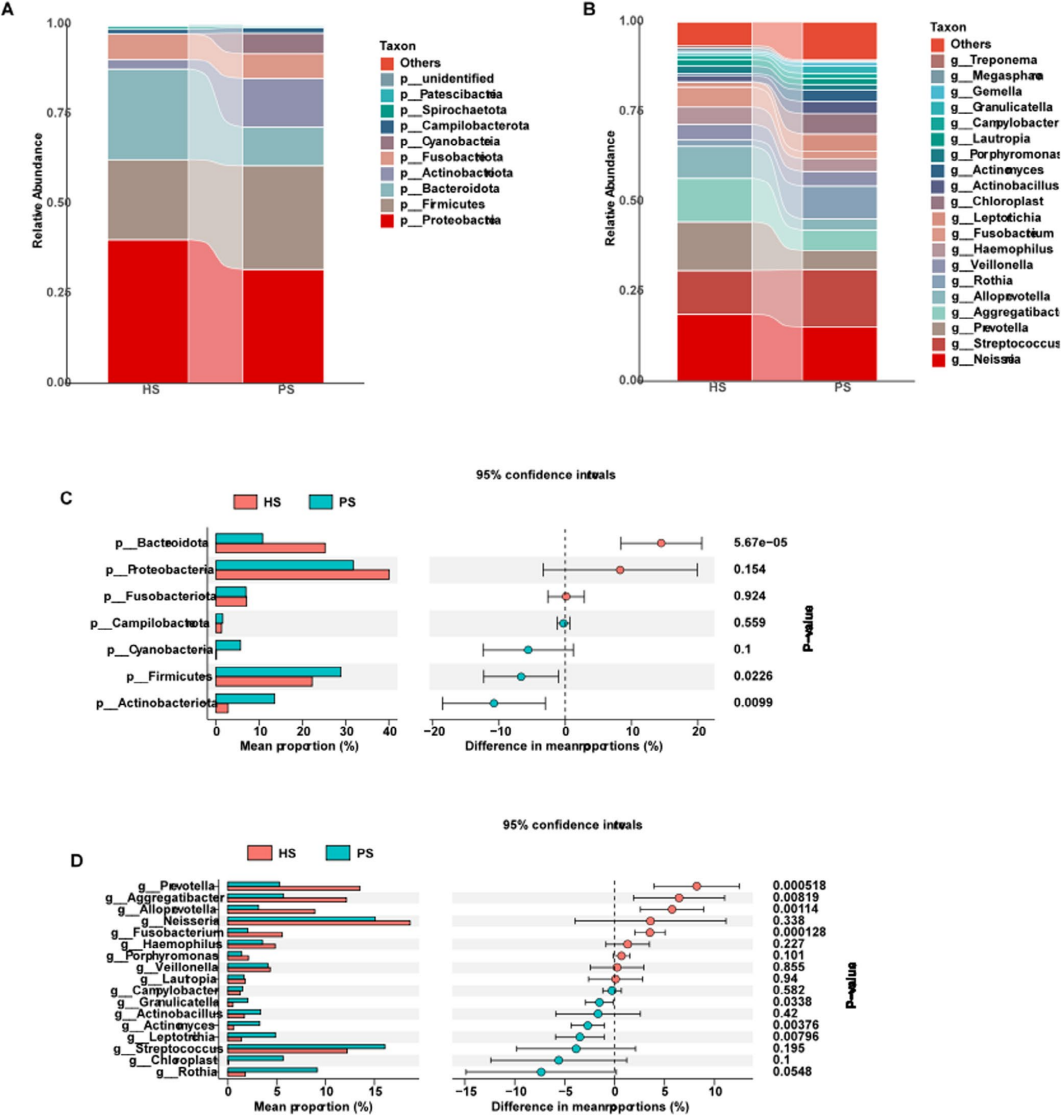
Microbial Community Composition and Differential Abundance Analysis between the two groups. PS group: Pancreatic neuroendocrine tumors; HS group: Healthy volunteers. (A) Stacked bar plot showing the relative abundance of bacterial phyla in the HS and PS groups. (B) Stacked bar plot showing the relative abundance of bacterial genera in the HS and PS groups. (C) Differential abundance analysis at the phylum level. (D) Differential abundance analysis at the genus level.

We particularly measured the differences in the taxa at the phylum and genus levels. At the phylum level, *Proteobacteria, Firmicutes, Bacteroidota, Actinobacteriota, Fusobacteriota, Cyanobacteria*, and *Campilobacterota* were the seven dominant oral flora in the two groups (Figure 3A). The relative abundance of *Firmicutes* (*p* = 0.023) and *Actinobacteriota* (*p* = 0.010) was higher in the PS group compared to the HS group, whereas *Bacteroidota* was higher in the HS group (*p* = 0.000) (Figure 3B).

In addition, at the genus level, the HS group was composed mainly of *Neisseria* (18.7%), *Streptococcus* (12.2%), *Prevotella* (13.5%), *Aggregatibacter* (12.1%), *Alloprevotella* (8.9%), and *Rothia* (1.8%), followed by *Veillonella* (4.3%), *Haemophilus* (4.9%), *Fusobacterium* (5.6%), *Leptotrichia* (1.4%), *Chloroplast* (0.1%), and *Actinobacillus* (1.3%) (Figure 3C). The relative abundance of *Prevotella, Aggregatibacter, Alloprevotella*, and *Fusobacterium* was higher in the HS group (*p* = 0.000, 0.008, 0.001, and 0.000, respectively), whereas the relative abundance of *Leptotrichia*, *Actinobacillus*, and *Granulicatella* was lower (*p* = 0.003, 0.007, and 0.033, respectively) (Figure 3D).

### 3.5 Characterized Oral Microbial Taxa Associated With pNETs Patients

**FIGURE 4:**
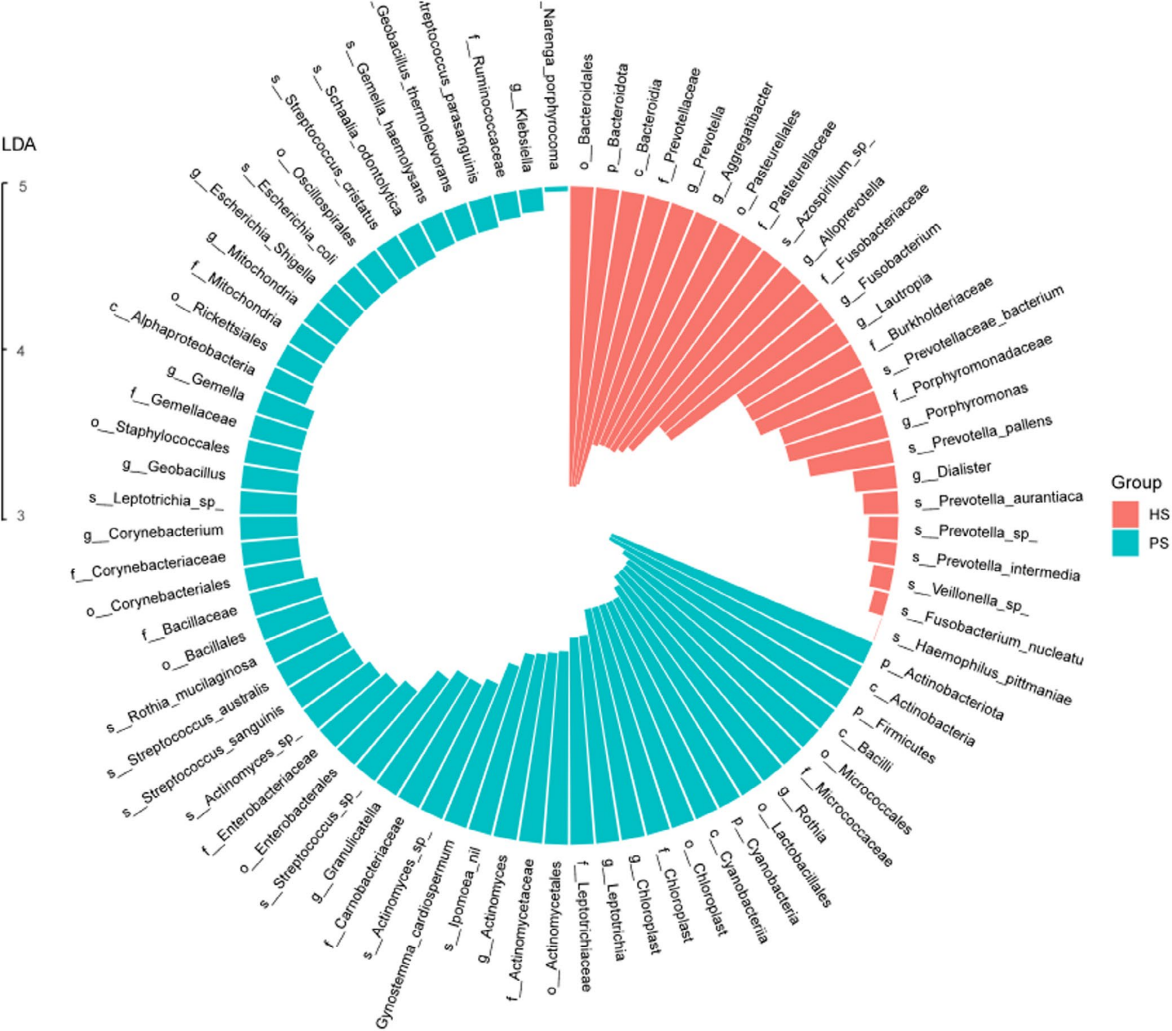
LEfSe analysis showed the differences in relative abundance at the phylum and genus levels between the two groups.

Taxa with linear discriminant analysis (LDA) values > 3.0 are depicted in Figure 4. The LEfSe cladogram directly shows these important microbial biomarkers for the groups in all taxa (Figure 4). Consequently, our analysis identified *Actinobacteriota*, *Actinobacteria*, *Firmicutes*, *Bacilli*, and *Micrococcales* as significant taxa responsible for the observed alterations in the microbiome of patients with pNETs, thus suggesting their potential utility as robust markers in the PS group. Conversely, *Bacteroidales*, *Bacteroidota*, *Bacteroidia*, *Prevotellaceae*, and *Prevotella* were identified as key taxa driving the changes in the microbiome composition of healthy controls. Furthermore, at the genus level, the top five biomarkers for the PS group were determined to be *Rothia*, *Chloroplast*, *Leptotrichia*, *Actinomyces*, and *Granulicatella* based on their differential abundance.

### 3.6 Functional Analysis of Oral Microbiota Between the Two Groups

Phylogenetic Investigation of Communities by Reconstruction of Unobserved States (PICRUSt) was conducted to predict the metagenomes and identify the KEGG pathways involved in each group.

**FIGURE 5:**
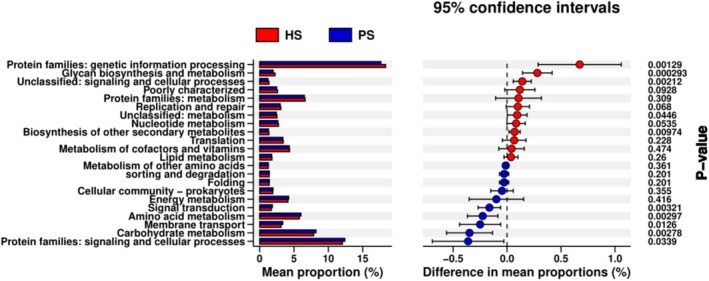
The function prediction of the two groups. PS group: Pancreatic neuroendocrine tumors; HS group: Healthy volunteers. Differential KEGG pathways were analyzed using PICRUSt for two groups. Significant differences between the PS group and the healthy volunteers, the HS group, were presented.

The predicted KEGG pathways significantly enriched in the PS group included signaling and cellular processes, carbohydrate metabolism, membrane transport, amino acid metabolism, and signal transduction, while there was a reduction of genes related to genetic information processing, glycan biosynthesis and metabolism, and biosynthesis of other secondary metabolites (Figure 5).

## 4 Discussion

pNETs represent a relatively rare subset, accounting for only 1% ~ 2% of pancreatic tumors [11]. However, there has been a progressive increase in their incidence in recent years [12]. These tumors can occur across all age groups. The World Health Organization (WHO) classifies pNETs into two main subtypes: Well‐differentiated pancreatic neuroendocrine tumors and the more aggressive poorly differentiated pancreatic neuroendocrine carcinomas [13]. Poorly differentiated pancreatic neuroendocrine carcinomas exhibit aggressive behavior and a high fatality rate, with the majority of patients succumbing within one year of diagnosis [14, 15], contrasting with the relatively indolent nature of well‐differentiated pNETs. Therefore, it is crucial to enhance early detection rates for pNETs and conduct research focused on their prevention and etiology. Despite extensive research, the precise origin of pNETs remains unknown.

Oral bacteria are established pathogenic agents in periodontal diseases and can induce systemic inflammation, having been linked to conditions such as diabetes, lung disease, and rheumatoid arthritis [16, 17]. A growing body of evidence further suggests a correlation between the oral microbiome and tumor development [18, 19, 20]. Notably, a study reported an 8.8% detection rate of *Clostridium* in pancreatic cancer cases, with higher cancer‐related mortality observed in the positive group. These findings suggest that *Clostridium* might be independently associated with a poor prognosis in pancreatic cancer and could serve as a prognostic biomarker [21]. Conversely, it has been reported that a higher level of antibodies to *Porphyromonas gingivalis* in serum is associated with a reduced risk of pancreatic cancer [22]. However, limited research exists on the relationship between oral flora and pancreatic neuroendocrine tumors. Considering this, we hypothesized that patients with pNETs harbor distinct oral microbial communities compared to healthy individuals. To test this, we conducted a comprehensive 16S rRNA gene sequencing analysis. Our results revealed a noticeable, though statistically non‐significant, increase in the abundance and diversity of the oral microbiota in the pNET group compared to healthy controls.

An intriguing finding is the discrepancy in microbial diversity patterns: While the HS group exhibited a greater number of unique OTUs, the PS group showed a trend toward higher alpha diversity. This may reflect a disease‐specific restructuring of the microbial community. The unique OTUs in the HS group likely represent rare and sensitive flora to the healthy oral niche, which contribute minimally to overall diversity indices. In contrast, the disease‐altered oral environment in the PS group may act as an ecological filter, eliminating some of these rare taxa while promoting the proliferation of certain bacterial groups adapted to the diseased state. This process could lead to a more homogenized community structure, thereby resulting in a numerically higher alpha diversity. These findings further suggest that the influence of disease on the microbiome involves shifts in the relative abundance of shared species and changes in community assembly, rather than a mere reduction in total species richness.

At the phylum level, patients with pNETs showed a significant increase in the relative abundance of Firmicutes and Actinobacteriota compared to healthy controls. Firmicutes represent a major component of the intestinal microbiota in humans and other mammals, comprising approximately 50%–60% of the total microbial community. This phylum has been linked to obesity due to its role in degrading insoluble dietary fiber and enhancing energy harvest. Previous studies indicate that an elevated Firmicutes‐to‐Bacteroides ratio contributes to diet‐induced obesity by improving the efficiency of energy extraction from food [23].

At the genus level, we observed a statistically significant increase in the relative abundance of Leptotrichia, Actinobacillus, and Granulicatella in the PS group compared to the HS group. Leptotrichia is an opportunistic pathogen implicated in periodontal disease, lung abscess, pneumonia, osteomyelitis, endocarditis, and bloodstream infections—particularly in immunocompromised individuals [24, 25, 26]. It can also elicit a systemic immune response, leading to detectable serum antibodies [24]. In the study conducted by Torres et al. [27], an increased proportion of *Leptotrichia* was observed in patients with pancreatic cancer. Similarly, our study identified a relationship between *Leptotrichia* and pNETs. *Actinobacillus, a gram‐negative bacillus within the Pasteuriaceae family, commonly colonizes the respiratory and urogenital mucosa of healthy animals but can cause invasive disease following local trauma* [28]. *Granulicatella, a gram‐positive facultative anaerobe, has been isolated from root canal infections and from the peritoneal fluid of gastric cancer patients* [29, 30]. In summary, we identify these three genera as potential biomarkers associated with pNETs.

Beyond taxonomic shifts, we also predicted functional differences between the two groups. Our analysis revealed elevated activity in pathways related to signaling and cellular processes, carbohydrate metabolism, membrane transport, amino acid metabolism, and signal transduction, suggesting potential functional mechanisms underlying pNET pathogenesis.

This study has several limitations. First, the small sample size—constrained by the low incidence of pNETs—limits statistical power and generalizability, and precludes stratified analysis of confounders such as age, sex, and smoking. Future multi‐center studies with larger cohorts are needed to address this. Second, although dietary guidance may have introduced some homogeneity, dietary patterns were not formally quantified or controlled. Future work should include detailed dietary assessments and, if needed, sensitivity analyses to isolate disease‐specific effects. Third, functional inferences rely on PICRUSt predictions from 16S data and require validation through metagenomic, metabolomic, or experimental approaches. Finally, while the standardized workflow ensures reproducibility, it does not represent a methodological innovation.

In summary, this study delineates oral microbial alterations associated with pNETs and proposes several microbial features for validation as potential biomarkers. These findings provide a meaningful foundation and clear targets for future research aimed at elucidating the role of oral microbiota in pNETs pathogenesis and evaluating its clinical translational potential.

## Author Contributions

**Jiayue Yao:** data curation, writing – original draft, investigation. **Chunming Fei:** data curation, writing – original draft, investigation. **Han Wu:** writing – original draft. **Zhenyu Zhang:** project administration, writing – review and editing, supervision, funding acquisition. **Zongdan Jiang:** project administration, writing – review and editing, supervision, funding acquisition.

## Funding

This work was supported by the Nanjing Health Science and Technology Development Special Fund Project Plan (YKK20108) and the Science and Technology Development Project of Nanjing Medical University (NMUB2019134).

## Ethics Statement

The study protocol was approved by the institutional review board of Nanjing Medical University, and all the experiments were performed in accordance with approved guidelines and regulations.

## Consent

Written informed consent was obtained from all participants or their guardians for the publication of their data.

## Conflicts of Interest

The authors declare no conflicts of interest.

## Data Availability

The data that support the findings of this study are openly available in NCBI Sequence Read Archive database at https://www.ncbi.nlm.nih.gov/sra, reference number PRJNA984637.
